# Postural Stability and Physical Fitness in Preschool Children: Associations with Lower-Limb Muscular Strength, Speed/Agility, and Cardiorespiratory Fitness

**DOI:** 10.3390/children13070910

**Published:** 2026-07-09

**Authors:** Andrés Godoy-Cumillaf, Josivaldo de Souza-Lima, Frano Giakoni-Ramírez, Catalina Muñoz-Strale, Maribel Parra-Saldias, Daniel Duclos-Bastias, Claudio Farias-Valenzuela, Eugenio Merellano-Navarro, José Bruneau-Chávez

**Affiliations:** 1 Grupo de Investigación en Educación Física, Salud y Calidad de Vida (EFISAL), Facultad de Educación, Universidad Autónoma de Chile, Temuco 4780000, Chile; andres.godoy@uautonoma.cl; 2 Facultad de Educación y Ciencias Sociales, Instituto del Deporte y Bienestar, Universidad Andres Bello, Las Condes, Santiago 7550000, Chile; josivaldo.desouza@unab.cl (J.d.S.-L.); frano.giakoni@unab.cl (F.G.-R.); catalina.munoz@unab.cl (C.M.-S.); 3 Departamento de Educación Física, Deporte y Recreación, Universidad de Atacama, Copiapó 1530000, Chile; maribel.parra@uda.cl; 4 iGEO, Escuela de Educación Física, Facultad de Filosofía y Educación, Pontificia Universidad Católica de Valparaíso, Valparaíso 2340025, Chile; 5 METIS Research Lab, Facultad de Negocios y Tecnología, Universidad Alfonso X el Sabio (UAX), 28691 Madrid, Spain; 6 Escuela de Ciencias de la Actividad Física, el Deporte y la Salud, Universidad de Santiago de Chile (USACH), Santiago 9170022, Chile; claudio.farias.v@usach.cl; 7 Department of Physical Activity Sciences, Faculty of Education Sciences, Universidad Catόlica del Maule, Talca 3530000, Chile; 8 Departamento de Educación Física, Deportes y Recreación, Universidad de la Frontera, Temuco 4811230, Chile; jose.bruneau@ufrontera.cl

**Keywords:** motor competence, balance control, early childhood, physical development, functional performance

## Abstract

**Highlights:**

**What are the main findings?**

- Greater postural stability was independently associated with better lower-limb muscular strength and cardiorespiratory fitness in preschool children, even after adjustment for age, sex, BMI, and object-control skills.
- The associations between postural stability and lower-limb muscular strength and cardiorespiratory fitness remained robust across multiple sensitivity analyses, whereas evidence for speed/agility was less consistent and no association was observed with flexibility.

**What are the implications of the main findings?**

- Postural stability may represent a useful functional marker of early physical development and could contribute to the identification of children with less favorable physical fitness profiles.
- Programs aimed at improving balance and postural control during the preschool years may have the potential to support the development of selected components of physical fitness, particularly lower-limb muscular strength and cardiorespiratory fitness.

**Abstract:**

**Background/Objectives**: Postural stability is considered a key component of motor development during early childhood; however, its specific association with different components of physical fitness in preschool children remains insufficiently explored. This study aimed to evaluate the association between postural stability and physical fitness components in preschool children. **Methods**: A cross-sectional study was conducted in 134 preschool children. Physical fitness was assessed through lower-limb muscular strength (standing long jump), speed/agility (4 × 10 m shuttle run), cardiorespiratory fitness (20 m shuttle run), and flexibility (sit-and-reach test). Object-control skills and postural stability were evaluated using selected tasks from the Movement Assessment Battery for Children-2. Composite indices were created for object-control skills and postural stability. Multiple linear regression analyses were performed adjusting for age, sex, body mass index (BMI), and object-control skills. Additional analyses included ANCOVA by postural stability tertiles and sensitivity analyses using HC3 robust standard errors, exclusion of influential observations, and bootstrap resampling. **Results**: Greater postural stability was independently associated with better lower-limb muscular strength (B = 9.49; 95% CI: 4.39–14.60; *p* ≤ 0.001) and cardiorespiratory fitness (B = 0.27; 95% CI: 0.06–0.49; *p* = 0.014). An association with speed/agility was observed in the primary model but lost statistical significance in sensitivity analyses. No significant association was found with flexibility. ANCOVA analyses confirmed significant differences across postural stability tertiles for lower-limb muscular strength (*p* = 0.033) and cardiorespiratory fitness (*p* = 0.011). Sensitivity analyses supported the robustness of the associations for lower-limb muscular strength and cardiorespiratory fitness. **Conclusions**: Greater postural stability was consistently associated with better lower-limb muscular strength and cardiorespiratory fitness in preschool children. These findings suggest that postural stability is associated with selected components of physical fitness during early childhood. Longitudinal and intervention studies are needed to clarify the direction and potential implications of these associations.

## 1. Introduction

The preschool period represents a critical stage for motor, neuromuscular, and functional development, as fundamental motor skills are consolidated, postural control patterns are refined, and important foundations are established for participation in physical activity and subsequent physical fitness development [1,2,3,4,5]. Recent evidence suggests that, in young children, motor competence, fundamental motor skills, and physical fitness should not be considered independent domains but rather closely interconnected dimensions of child development [2,4,5,6,7,8].

In this context, physical fitness in preschool children has received increasing attention because of its relationship with current and future health outcomes. Recent reviews have shown that field-based tests such as the standing long jump, speed/agility runs, flexibility assessments, balance tests, and the shuttle run are widely used to evaluate relevant components of physical fitness during this developmental stage, reinforcing their utility in both research and applied settings [1]. Furthermore, better physical fitness during the preschool years has been associated with more favorable adiposity profiles, motor performance, cognitive functioning, and overall health, highlighting the importance of identifying its early determinants [3,6,9,10,11,12].

Fundamental motor skills also play a central role in early development. They are commonly classified into locomotor, object-control, and stability skills. Recent literature has shown that higher levels of fundamental motor skills are associated with more favorable indicators of physical activity, motor performance, and physical fitness, suggesting that their adequate development may contribute to healthier developmental trajectories from an early age [2,4,5,7,8,13]. Object-control skills, such as catching and throwing, have demonstrated relevant associations with motor performance and several components of physical fitness in preschool populations [7,13].

Motor competence is widely recognized as a multidimensional construct comprising several interrelated domains, including postural stability, locomotor skills, and object-control skills [13,14]. Although these domains contribute jointly to motor development, they rely on partly distinct, although interrelated, neuromuscular and biomechanical processes and may therefore show different relationships with components of physical fitness [14,15]. Accordingly, examining postural stability separately from object-control skills may provide a more specific understanding of its independent contribution to physical fitness during early childhood.

Within this framework, postural stability represents a particularly relevant dimension of motor development. Postural control enables individuals to maintain and adjust body position in response to both static and dynamic demands and depends on the integration of visual, vestibular, and somatosensory information together with coordinated muscular responses [16,17]. During the preschool years, these systems are still undergoing maturation, and therefore individual differences in postural stability may translate into meaningful differences in motor and physical performance [16,17,18,19]. Moreover, recent systematic reviews have highlighted the importance of balance and postural control as clinically useful and methodologically relevant constructs in pediatric research [16,17].

Despite these advances, important gaps remain in the literature. A considerable proportion of recent studies have focused on the relationships among physical activity, global motor competence, and physical fitness, whereas the specific association between postural stability and individual components of physical fitness in preschool children has received comparatively less attention [2,4,5,8,13]. Evidence remains limited regarding whether postural stability is independently associated with lower-limb muscular strength, speed/agility, cardiorespiratory fitness, and flexibility while accounting for potential confounding variables such as age, sex, body mass index, and object-control skills [3,7,13]. Addressing this gap is important because a better understanding of these relationships may contribute to optimizing early motor assessment and the design of interventions aimed at promoting physical development during early childhood [2,5,8,13].

From an applied perspective, identifying associations between postural stability and physical fitness during the preschool years may provide useful information for physical education programs, physical activity promotion strategies, and the early identification of children with less favorable motor profiles. Given that this developmental stage is considered a key window for the acquisition of fundamental motor skills and other essential developmental processes, understanding these relationships may have important implications for health promotion and motor functioning later in life [4,6,10,20].

Therefore, the aim of the present study was to evaluate the association between postural stability and physical fitness components in preschool children. We hypothesized that children with greater postural stability would exhibit better lower-limb muscular strength, speed/agility, and cardiorespiratory fitness, independently of age, sex, body mass index, and object-control skills. Flexibility was included as an exploratory outcome because evidence regarding its association with postural stability in preschool children remains limited and inconsistent.

## 2. Materials and Methods

### 2.1. Study Design and Participants

A cross-sectional observational study was conducted in preschool children aged 4–6 years attending seven preschools in Temuco, Chile. The study employed a convenience sampling approach. After obtaining authorization from the directors of the participating preschools, all eligible children enrolled at these centers (approximately 210 children) were invited to participate. Parents or legal guardians received detailed information about the study and provided written informed consent prior to participation. In addition, verbal assent was obtained from all children immediately before the assessments.

Children were eligible if they were between 4 and 6 years of age, were enrolled in one of the participating preschools, and had written parental consent together with verbal child assent. Children presenting motor limitations that prevented them from completing the physical assessments were excluded from participation.

A total of 154 children completed the assessment protocol. Of these, 134 had complete data for all variables included in the present analyses and therefore comprised the final analytical sample. Information was collected on anthropometric characteristics, physical fitness, object-control skills, and postural stability. All assessments were performed by trained evaluators following standardized procedures.

The study was conducted in accordance with the ethical principles for research involving human participants and the Declaration of Helsinki. All procedures were reviewed and approved by the Ethics Committee of Universidad Autónoma de Chile (CEC-N°31-22), with approval granted on 8 August 2022.

### 2.2. Anthropometric Assessment

Age and sex were recorded for all participants. Body weight was measured using a calibrated digital scale, and height was assessed using a portable stadiometer. Participants were evaluated barefoot and wearing light clothing. Body mass index (BMI) was subsequently calculated as weight divided by height squared (kg/m^2^). BMI was treated as a continuous variable in all statistical analyses and was not categorized according to age- or sex-specific reference standards.

### 2.3. Physical Fitness Assessment

Physical fitness was primarily assessed using field-based tests included in the PREFIT battery [21], an instrument that has demonstrated feasibility, reliability, and suitability for use in preschool children [22]. The battery has been previously applied in Chilean preschool populations [23,24], and its reliability has recently been confirmed in Chilean preschoolers [25]. In addition, flexibility was assessed using the sit-and-reach test following standardized procedures. The following components of physical fitness were evaluated: Lower-limb muscular strength (LLMS) assessed using the standing long jump test. Participants were instructed to jump horizontally as far as possible from a standing position using both feet simultaneously. Speed/agility assessed using the 4 × 10 m shuttle run test. Participants completed four consecutive 10-m runs as quickly as possible, and total completion time was recorded. Cardiorespiratory fitness (CRF) assessed using the 20 m shuttle run test. Participants ran back and forth between two lines 20 m apart following externally paced audio signals until exhaustion or inability to maintain the required pace. Flexibility assessed using the sit-and-reach test. Participants performed a forward trunk flexion from a seated position with knees fully extended, and the furthest distance reached was recorded.

To minimize measurement error, the recommendations proposed for the PREFIT battery were followed, including the sequence of test administration, the use of comfortable sports clothing and appropriate footwear, and the provision of standardized encouragement and motivation adapted to the age of the participants [21].

### 2.4. Motor Competence Assessment

Motor competence was assessed using selected tasks from the Movement Assessment Battery for Children, second edition (MABC-2) [26], which has demonstrated adequate psychometric properties, including acceptable reliability and reproducibility in pediatric populations [27].

The assessment included tasks representing two domains of motor competence. Object-control skills were evaluated through the catching and aiming/throwing tasks, which require visuomotor coordination, upper-limb control, and object manipulation, and are widely used to assess this domain of motor competence in preschool children [26,27].

Postural stability was evaluated through four balance-related tasks derived from the MABC-2 assessment: right-leg balance, left-leg balance, tiptoe walking, and floor mat tasks. These tasks were selected because they assess complementary aspects of postural stability, including unilateral static balance, dynamic balance during gait, and balance under altered sensory conditions. Together, they provide a broader representation of balance performance than any individual task alone while remaining conceptually focused on postural stability.

Performance in each task was scored according to the procedures established in the MABC-2 manual [26].

### 2.5. Construction of Composite Variables

Two composite indices were created using standardized scores (z-scores). Before standardization, the direction of all variables was verified to ensure that higher scores consistently represented better performance. For the speed/agility test, the inverse nature of the outcome was considered during interpretation, as lower completion times indicate better performance.

For each task, standardized scores were calculated by subtracting the sample mean from each individual value and dividing by the sample standard deviation. The Object Control Index (OCI) was calculated as the average of the standardized scores obtained in the catching and throwing tasks. The Postural Stability Index (PSI) was calculated as the average of the standardized scores obtained in right-leg balance, left-leg balance, tiptoe walking, and floor mat tasks. Because the PSI was calculated as the average of standardized scores, a one-unit increase represents an increase of approximately one standard deviation in the composite postural stability score relative to the study sample.

The four tasks included in the PSI were selected because they assess complementary aspects of postural stability, including unilateral static balance, dynamic balance during gait, and balance under altered sensory conditions. Rather than representing interchangeable measures, these tasks capture related but distinct manifestations of balance performance. To evaluate the coherence of the composite index, internal consistency was assessed using Cronbach’s alpha and Pearson correlations among the component tasks.

### 2.6. Statistical Analysis

Continuous variables were described as mean and standard deviation when normally distributed, or as median and interquartile range when normality assumptions were not met. Categorical variables were presented as absolute frequencies and percentages. Normality of continuous variables was assessed using the Shapiro–Wilk test.

To evaluate the association between postural stability and physical fitness, multiple linear regression models were fitted using lower-limb muscular strength (LLMS), speed/agility, cardiorespiratory fitness (CRF), and flexibility as dependent variables. Age, sex, body mass index (BMI), OCI, and PSI were included as independent variables in all models. Sex was entered into the regression models as a binary variable (0 = female, 1 = male). Object-control skills (OCI) were included as an adjustment variable to estimate the independent association between postural stability and physical fitness outcomes while reducing potential confounding arising from another important domain of motor competence. Results were reported as unstandardized coefficients (B), standard errors (SE), standardized coefficients (β), 95% confidence intervals (95% CI), *p*-values, and adjusted coefficients of determination (adjusted R^2^).

As a complementary analysis, PSI was categorized into tertiles representing low, medium, and high postural stability. Analyses of covariance (ANCOVA) were subsequently performed for LLMS, speed/agility, CRF, and flexibility, using postural stability tertiles as the fixed factor and adjusting for age, sex, BMI, and OCI. Adjusted means, 95% confidence intervals, global *p*-values, partial eta-squared (η^2^p) effect sizes, and Bonferroni-adjusted post hoc comparisons were reported.

Regression assumptions were evaluated for all models. Residual normality was assessed using the Shapiro–Wilk test, homoscedasticity using the Breusch–Pagan test, multicollinearity using variance inflation factors (VIF), and influential observations using Cook’s distance.

As sensitivity analyses, the primary regression models were repeated using HC3 heteroscedasticity-consistent standard errors, after excluding influential observations defined as Cook’s distance values greater than 4/n for each model and using bootstrap resampling with 2000 iterations to estimate confidence intervals for the PSI regression coefficients.

Statistical significance was established at *p* < 0.05. All analyses were performed using R statistical software (version 4.6.0; R Foundation for Statistical Computing, Vienna, Austria).

## 3. Results

A total of 134 preschool children were included, of whom 76 were girls (56.7%) and 58 were boys (43.3%). Median age was 6.0 years (IQR: 5.0–6.0), and median BMI was 17.35 kg/m^2^ (IQR: 15.91–18.71). According to the Shapiro–Wilk test, lower-limb muscular strength and Flexibility showed approximately normal distributions, whereas Age, BMI, speed/agility, cardiorespiratory fitness, Object Control Index (OCI), and Postural Stability Index (PSI) deviated from normality. Complete sample characteristics are presented in Table 1.

Before examining the associations with physical fitness outcomes, the internal consistency of the Postural Stability Index (PSI) was evaluated. The four tasks included in the PSI showed moderate internal consistency (Cronbach’s α = 0.62). Pearson correlations ranged from 0.25 to 0.67, with the strongest association observed between right- and left-leg balance (r = 0.67). These findings provide empirical support for the use of the composite index while indicating that the included tasks capture complementary aspects of postural stability rather than identical manifestations of balance performance (Supplementary Table S1).

In the multiple linear regression models adjusted for age, sex, BMI, and OCI, PSI was significantly associated with better performance in lower-limb muscular strength, speed/agility, and cardiorespiratory fitness, but not with Flexibility (Table 2).

For lower-limb muscular strength, higher PSI was associated with greater jump distance (B = 9.49, 95% CI: 4.39 to 14.60, *p* ≤ 0.001). Age and OCI were positively associated with performance, whereas BMI showed an inverse association. The model explained 40.9% of the observed variance.

For speed/agility, higher PSI was associated with lower completion time in the primary analysis (B = −0.50, 95% CI: −0.97 to −0.03, *p* = 0.036), indicating better performance. Significant associations were also observed for Age and OCI. The model explained 30.8% of the variance.

For cardiorespiratory fitness, PSI was positively associated with cardiorespiratory fitness performance (B = 0.27, 95% CI: 0.06 to 0.49, *p* = 0.014), independently of Age, Sex, BMI, and OCI. Age was positively associated with performance, whereas BMI showed an inverse association. The model explained 34.5% of the variance.

In contrast, PSI was not significantly associated with flexibility (B = 1.04, 95% CI: −0.48 to 2.56, *p* = 0.177). This model showed limited explanatory capacity (adjusted R^2^ = 0.086).

Overall, these findings indicate that greater postural stability was associated with better lower-limb muscular strength and cardiorespiratory fitness. Evidence for speed/agility was initially favorable but less consistent in subsequent sensitivity analyses. Standardized coefficients further indicated that PSI was among the strongest independent predictors of lower-limb muscular strength and cardiorespiratory fitness performance.

When PSI was categorized into tertiles and analyses were adjusted for Age, Sex, BMI, and OCI (Table 3), significant overall differences were observed for lower-limb muscular strength (*p* = 0.033; partial η^2^ = 0.052) and cardiorespiratory fitness (*p* = 0.011; partial η^2^ = 0.068), whereas no significant differences were found for speed/agility or flexibility.

For lower-limb muscular strength, adjusted means were 73.09, 81.46, and 84.58 in the low-, medium-, and high-stability tertiles, respectively. Complete adjusted means and 95% confidence intervals for all outcomes are provided in Supplementary Table S2. Bonferroni-adjusted post hoc analyses revealed significant differences between the low- and high-stability tertiles (*p* = 0.032). Full pairwise comparisons are presented in Supplementary Table S3.

**Figure 1:**
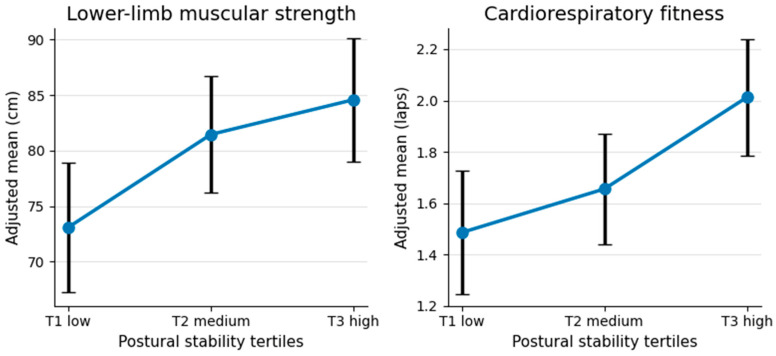
Adjusted means (95% confidence intervals) for lower-limb muscular strength (standing long jump) and cardiorespiratory fitness (20-m shuttle run) according to postural stability tertiles (T1 = low, T2 = medium, T3 = high). Values represent adjusted means estimated from ANCOVA models adjusted for age, sex, body mass index, and object-control skills. Error bars represent 95% confidence intervals.

For cardiorespiratory fitness, adjusted means were 1.49, 1.66, and 2.01 in the low-, medium-, and high-stability tertiles, respectively. A significant difference was again observed between the low- and high-stability tertiles (*p* = 0.013). Adjusted means for outcomes showing significant differences are illustrated in Figure 1.

Detailed diagnostic analyses are presented in Supplementary Table S4. Overall, no relevant multicollinearity was detected, with low variance inflation factor values across all models. However, some deviations from residual normality were identified, and evidence of heteroscedasticity was observed in the lower-limb muscular strength, cardiorespiratory fitness, and flexibility models. In addition, a limited number of potentially influential observations were identified according to Cook’s distance.

Sensitivity analyses demonstrated that the association between PSI and lower-limb muscular strength remained statistically significant across all model specifications, including HC3 robust standard errors, exclusion of influential observations, and bootstrap analyses. Similarly, the association between PSI and cardiorespiratory fitness remained statistically significant across all sensitivity analyses and became slightly stronger after exclusion of influential observations.

In contrast, the association between PSI and speed/agility lost statistical significance when robust standard errors were applied and after influential observations were excluded, suggesting reduced robustness of this finding. No consistent associations were observed between PSI and flexibility across any sensitivity analysis (Table 4). Bootstrap results are presented in Supplementary Table S5 and were consistent with the primary findings.

Overall, sensitivity analyses support the consistency of the associations between postural stability and both lower-limb muscular strength and cardiorespiratory fitness.

## 4. Discussion

The main findings of this study indicate that greater postural stability was independently associated with better lower-limb muscular strength (LLMS) and cardiorespiratory fitness (CRF) in preschool children, even after adjusting for age, sex, BMI, and object-control skills. Moreover, these associations remained statistically significant across all sensitivity analyses, including HC3 robust standard errors, exclusion of influential observations, and bootstrap resampling, supporting the robustness of the findings. In contrast, the association observed with speed/agility in the primary model was less stable, as it lost statistical significance in the robustness analyses, whereas no clear association was observed with flexibility. Taken together, these findings suggest that postural stability may be more closely related to physical fitness components that depend on efficient force production, force transmission, and whole-body motor control, such as lower-limb muscular strength and cardiorespiratory fitness, than to other components such as flexibility [9,11,16,18,28]. The interpretation of these findings should also consider the composite nature of the Postural Stability Index. The moderate internal consistency observed for the PSI (Cronbach’s α = 0.62) is consistent with the expectation that its component tasks assess related but not identical aspects of balance performance. Accordingly, the composite index was intended to provide a broader representation of postural stability in preschool children than any individual balance task alone.

From a physiological and biomechanical perspective, several plausible mechanisms have been proposed to explain the observed associations. Postural control may constitute a functional foundation for the efficient execution of motor tasks because it may facilitate body alignment, stabilization of proximal segments, and optimization of force transmission during activities such as jumping, running, and changing direction [16,17,18,19,28]. In young children, who are still undergoing active neuromuscular maturation, a greater ability to control the center of mass may facilitate force production and improve the mechanical efficiency of whole-body movements [16,18,19,28]. Consequently, the observed association with LLMS may reflect the importance of postural control for the generation and transfer of force during explosive lower-extremity actions, such as those required in the standing long jump test. Similarly, the association observed with CRF may not exclusively reflect superior cardiorespiratory capacity but could also be related to a more efficient execution of an intermittent task requiring accelerations, decelerations, changes in direction, and dynamic body control [1,9,11,29]. In contrast, the absence of a consistent association with flexibility may be explained by the fact that this component depends more strongly on structural and anatomical factors, and its functional contribution to postural control may be less direct than that of muscular strength, coordination, or segmental stability [1,16]. Although these mechanisms are supported by previous literature, they should be regarded as plausible explanations rather than mechanisms demonstrated by the present cross-sectional study.

One noteworthy finding was the instability of the association observed for speed/agility. Although PSI was significantly associated with speed/agility in the primary regression model, this relationship was no longer statistically significant after applying robust estimators and excluding influential observations. This suggests that the association may be weaker and more sensitive to sample-specific characteristics than those observed for LLMS and CRF. Speed/agility performance in preschool children is likely influenced by multiple factors, including coordination, movement technique, reaction ability, motivation, and task familiarity, which may reduce the relative contribution of postural stability alone to overall performance [30].

Our findings are consistent with previous studies reporting positive associations between global motor competence and physical fitness in preschool children [4,5,7,8,13,21]. In this regard, the present study extends existing evidence by showing that even when specifically focusing on postural stability and controlling for object-control skills, meaningful associations persist with selected components of physical fitness. Recent studies have also shown that balance, coordination, and other dimensions of non-aerobic fitness are related to different aspects of child development, suggesting the existence of shared functional mechanisms linking motor control and physical performance [9,10,11,12,31]. Furthermore, our findings are consistent with evidence highlighting the influence of adiposity on physical performance during early childhood, particularly in tasks requiring body displacement or projection, which aligns with the inverse associations observed between BMI and both LLMS and CRF [3,12,31].

From an applied perspective, these findings suggest that postural stability may represent a relevant functional marker of early physical development. In educational and community settings, the assessment of balance and postural control may help characterize children’s motor profiles, particularly regarding lower-limb muscular strength and cardiorespiratory fitness [9,18,21]. However, because the present study was cross-sectional, these findings should not be interpreted as evidence that improving postural stability will necessarily lead to improvements in physical fitness. Rather, if future longitudinal and intervention studies confirm these associations, programs targeting early motor development could consider incorporating activities aimed at improving balance, stability, and postural control to evaluate their potential contribution to different components of physical fitness [2,5,6,20,31].

This study has several strengths. First, multiple components of physical fitness were evaluated simultaneously together with measures of postural stability and object-control skills, allowing the examination of specific associations beyond a global measure of motor competence. Second, the construction of composite indices representing object-control skills (OCI) and postural stability (PSI) enabled a more integrated assessment of these domains. Additional strengths include adjustment for relevant covariates, complementary analyses based on postural stability tertiles, and the use of sensitivity analyses incorporating robust standard errors, exclusion of influential observations, and bootstrap procedures, all of which enhanced the analytical robustness of the findings.

However, several limitations should be considered when interpreting the results. First, the cross-sectional design precludes causal inference [4,8,13]. Second, the sample was recruited from a specific context, which may limit the generalizability of the findings. Third, some models showed deviations from residual normality and evidence of heteroscedasticity, although these issues were addressed through robustness analyses. Fourth, Fourth, PSI was derived from field-based balance assessments and may not fully capture all dimensions of postural control that could be evaluated using laboratory-based instruments. In addition, performance on field-based assessments in preschool children may be influenced by factors such as motivation, attention, task understanding, and familiarity with the testing procedures, which could contribute to measurement variability. Finally, the association observed for speed/agility was sensitive to model specification and therefore should be interpreted with greater caution than the associations observed for LLMS and CRF.

## 5. Conclusions

In conclusion, greater postural stability was consistently associated with better lower-limb muscular strength and cardiorespiratory fitness in preschool children, independently of age, sex, BMI, and object-control skills. These associations remained robust across multiple sensitivity analyses, whereas the evidence for speed/agility was less consistent and no significant association was observed with flexibility.

These findings suggest that postural stability is associated with selected components of physical fitness during early childhood. However, longitudinal and intervention studies are needed to clarify the direction and potential implications of these associations.

## Figures and Tables

**Table 1:** Sample characteristics (n = 134).

Variable	Descriptive Statistics	Shapiro–Wilk *p*-Value
Age (years)	6.0 (5.0–6.0)	<0.001
Sex	Girls: 76 (56.7%); Boys: 58 (43.3%)	-
BMI (kg/m^2^)	17.35 (15.91–18.71)	<0.001
Lower-limb muscular strength (cm)	79.7 ± 21.97	0.173
Speed/agility (s)	16.86 (15.81–18.28)	<0.001
Flexibility (cm)	30.55 ± 5.27	0.068
Cardiorespiratory fitness (stage *)	1.5 (1.09–2.2)	<0.001
OCI	0.06 (−0.42–0.47)	<0.001
PSI	0.06 (−0.49–0.56)	<0.001

* 1 stage = 1 min. Data are presented as mean ± standard deviation or median (interquartile range) according to distribution. BMI: body mass index; OCI: Object Control Index; PSI: Postural Stability Index.

**Table 2:** Multiple linear regression analyses examining the association between postural stability and physical fitness outcomes.

Outcome	Predictor	B	SE	β	95% CI		*p*	Adjusted R^2^
LLMS	Age	7.549	2.175	0.272	3.244	11.854	<0.001	0.409
	Sex	6.257	3.288	0.141	−0.249	12.764	0.059	0.409
	BMI	−1.402	0.570	−0.172	−2.530	−0.274	0.015	0.409
	OCI	6.775	2.414	0.238	1.997	11.552	0.005	0.409
	PSI	9.494	2.577	0.309	4.393	14.595	<0.001	0.409
Speed/agility	Age	−0.678	0.199	−0.288	−1.073	−0.282	<0.001	0.307
	Sex	−0.357	0.302	−0.095	−0.955	0.240	0.239	0.307
	BMI	0.033	0.052	0.047	−0.070	0.136	0.529	0.307
	OCI	−0.594	0.221	−0.246	−1.033	−0.155	0.008	0.307
	PSI	−0.502	0.236	−0.192	−0.970	−0.033	0.036	0.307
CRF	Age	0.418	0.092	0.375	0.236	0.601	<0.001	0.344
	Sex	−0.093	0.139	−0.052	−0.369	0.182	0.504	0.344
	BMI	−0.078	0.024	−0.238	−0.126	−0.030	0.001	0.344
	OCI	0.188	0.102	0.164	−0.014	0.391	0.067	0.344
	PSI	0.271	0.109	0.219	0.055	0.487	0.014	0.344
Flexibility	Age	1.304	0.648	0.196	0.020	2.588	0.046	0.085
	Sex	−0.645	0.980	−0.060	−2.585	1.295	0.511	0.085
	BMI	−0.066	0.170	−0.034	−0.403	0.269	0.695	0.085
	OCI	0.681	0.720	0.099	−0.743	2.106	0.345	0.085
	PSI	1.042	0.768	0.141	−0.478	2.564	0.177	0.085

Models adjusted for age, sex, BMI, and Object Control Index (OCI). B = unstandardized coefficient; SE = standard error; β = standardized coefficient; CI = confidence interval; BMI = body mass index; OCI = Object Control Index; PSI = Postural Stability Index; LLMS = Lower-Limb Muscular Strength; CRF = Cardiorespiratory Fitness.

**Table 3:** Analysis of covariance according to tertiles of postural stability.

Outcome	F	*p*	Partial η^2^
Lower-limb muscular strength	3.506	0.033	0.052
Speed/agility	1.741	0.179	0.026
Cardiorespiratory fitness	4.651	0.011	0.068
Flexibility	0.205	0.814	0.003

Models adjusted for age, sex, BMI, and Object Control Index (OCI). Partial η^2^ values represent effect sizes. Adjusted means and confidence intervals are provided in Supplementary Table S2. Bonferroni-adjusted pairwise comparisons are presented in Supplementary Table S2.

**Table 4:** Sensitivity analyses of the association between Postural Stability Index and physical fitness outcomes.

Outcome	OLS B (95% CI)	*p*	HC3 B (95% CI)	*p*	No Influential Cases B (95% CI)	*p*	HC3 + No Influential Cases B (95% CI)	*p*
LLMS	9.49 (4.39–14.60)	0.003	9.49 (4.77–14.22)	0.001	8.21 (3.25–13.17)	0.001	8.21 (3.33–13.09)	0.001
Speed/Agility	−0.50 (−0.97–−0.03)	0.036	−0.50 (−1.09–0.09)	0.096	−0.18 (−0.64–0.29)	0.450	−0.18 (−0.69–0.33)	0.493
CRF	0.27 (0.06–0.49)	0.014	0.27 (0.07–0.47)	0.008	0.31 (0.12–0.50)	0.001	0.31 (0.16–0.47)	0.002
Flexibility	1.04 (−0.48–2.56)	0.177	1.04 (−0.23–2.32)	0.107	0.80 (−0.53–2.14)	0.238	0.80 (−0.32–1.92)	0.158

Values represent the unstandardized regression coefficient (B) for the association between Postural Stability Index (PSI) and each physical fitness outcome. Models were adjusted for age, sex, BMI, and Object Control Index (OCI). OLS = ordinary least squares regression; HC3 = heteroscedasticity-consistent standard errors (type HC3); influential observations were identified using Cook’s distance > 4/n. BMI = body mass index; OCI = Object Control Index; PSI = Postural Stability Index; LLMS = Lower-Limb Muscular Strength; CRF = Cardiorespiratory Fitness.

## Data Availability

The data presented in this study are available on request from the corresponding author. The data are not publicly available due to ethical standards.

## Supplementary Materials

The following supporting information can be downloaded at: https://www.mdpi.com/article/10.3390/children13070910/s1, Table S1: Internal consistency and Pearson correlations among the tasks comprising the Postural Stability Index; Table S2: Adjusted mean and 95% confidence intervals for physical fitness outcomes according to tertiles of postural stability; Table S3: Bonferroni-adjusted post hoc comparison according to tertiles of postural stability; Table S4: Diagnostic assessment of regression model assumptions; Table S5: Bootstrap analyses of the association between Postural Stability Index and physical fitness outcomes.

## Abbreviations

The following abbreviations are used in this manuscript:

ANCOVA	Analysis of Covariance
BMI	Body Mass Index
CI	Confidence Interval
CRF	Cardiorespiratory Fitness
HC3	Heteroscedasticity-Consistent Standard Errors (Type HC3)
LLMS	Lower-Limb Muscular Strength
MABC-2	Movement Assessment Battery for Children, Second Edition
OCI	Object Control Index
OLS	Ordinary Least Squares
PSI	Postural Stability Index
PREFIT	Assessing FITness in PREschoolers Battery
SE	Standard Error
VIF	Variance Inflation Factor
