# Supplementary material for: Postural Stability and Physical Fitness in Preschool Children: Associations with Lower-Limb Muscular Strength, Speed/Agility, and Cardiorespiratory Fitness
Source: Children (Basel). 2026 Jul 9;13(7):910. doi: 10.3390/children13070910 (PMC13407253; doi:10.3390/children13070910)
Supplement: Supplementary file 1 [file children-13-00910-s001.zip › children-4410146-supplementary.pdf]

Table S1. Internal consistency and Pearson correlations among the tasks comprising the Postural Stability Index.

Outcome	Right-leg balance	Left-leg balance	Tiptoe walking	Floor mat
Right-leg balance	1.000	0.672	0.327	0.265
Left-leg balance	0.672	1.000	0.287	0.248
Tiptoe walking	0.327	0.287	1.000	0.315
Floor mat	0.265	0.248	0.315	1.000

Pearson correlations were calculated using complete cases. Cronbach's α for the four-item Postural Stability Index was 0.62.

Table S2. Adjusted mean and 95% confidence intervals for physical fitness outcomes according to tertiles of postural stability

Outcome	Tertile	Adjusted mean	95% CI Lower	95% CI Upper	Global p-value	Partial η^2
LLMS	T1	73.092	67.237	78.947	0.033	0.052
	T2	81.455	76.219	86.691	0.033	0.052
	T3	84.582	79.024	90.140	0.033	0.052
Speed/agility	T1	17.597	17.069	18.124	0.179	0.026
	T2	17.135	16.663	17.606	0.179	0.026
	T3	16.851	16.351	17.352	0.179	0.026
CRF	T1	1.485	1.246	1.725	0.011	0.068
	T2	1.655	1.441	1.870	0.011	0.068
	T3	2.012	1.784	2.239	0.011	0.068
Flexibility	T1	30.182	28.467	31.897	0.814	0.003
	T2	30.487	28.953	32.021	0.814	0.003
	T3	30.990	29.361	32.618	0.814	0.003

Values represent adjusted means and 95% confidence intervals derived from ANCOVA models adjusted for age, sex, BMI, and Object Control Index (OCI). T1 = low postural stability; T2 = medium postural stability; T3 = high postural stability. BMI = body mass index; OCI = Object Control Index.

Table S3. Bonferroni-adjusted post hoc comparison according to tertiles of postural stability

Outcome	Comparison 1	Comparison 2	Adjusted difference	Bonferroni-adjusted p-value
LLMS	T1	T2	-8.363	0.130
	T1	T3	-11.489	0.032
	T2	T3	-3.126	1.000
Speed/agility	T1	T2	0.461	0.639
	T1	T3	0.745	0.195
	T2	T3	0.283	1.000
CRF	T1	T2	-0.169	0.942
	T1	T3	-0.526	0.013
	T2	T3	-0.356	0.066
Flexibility	T1	T2	-0.305	1.000
	T1	T3	-0.808	1.000
	T2	T3	-0.502	1.000

Pairwise comparisons were adjusted using the Bonferroni correction. T1 = low postural stability; T2 = medium postural stability; T3 = high postural stability.

Table S4. Diagnostic assessment of regression model assumptions

Outcome	Shapiro–Wilk p-values (residuals)	Breusch-Pagan p-value	Maximum VIF	Number of influential cases (Cook´s distance > 4/n)	Maximum Cook´s distance
LLMS	0.018	0.565	1.621	5	0.047
Speed/agility	0.031	0.110	1.621	10	0.231
CRF	0.004	0.026	1.621	8	0.057
Flexibility	0.131	0.028	1.621	5	0.254

Residual normality was assessed using the Shapiro–Wilk test. Homoscedasticity was evaluated using the Breusch–Pagan test. Multicollinearity was assessed using variance inflation factors (VIF). Influential observations were identified using Cook's distance > 4/n.

Table S5. Bootstrap analyses of the association between Postural Stability Index and physical fitness outcomes.

Outcome	Valid Bootstrap Samples	Mean bootstrap Coefficient (B)	95% Bootstrap CI Lower	95% Bootstrap CI Upper	Proportion with Same Sign
LLMS	2000	9.390	4.543	13.877	0.999
Speed/agility	2000	-0.479	-0.989	0.109	0.955
CRF	2000	0.270	0.064	0.479	0.994
Flexibility	2000	1.003	-0.340	2.225	0.939

Bootstrap analyses were based on 2,000 resamples. B represents the mean bootstrap regression coefficient for the association between Postural Stability Index (PSI) and each physical fitness outcome. CI = confidence interval.
